# PREVALENCE AND CORRELATES OF DEPRESSION IN OLDER ADULTS: FINDINGS FROM THE LONGROAD STUDY

**DOI:** 10.1093/geroni/igad104.3534

**Published:** 2023-12-21

**Authors:** Yitao Xi

**Affiliations:** Columbia University, New York City, New York, United States

## Abstract

As an important public health issue, depression has been studied extensively in children and young adults. Epidemiologic data on depression for older adults are scant. The Longitudinal Research on Aging Drivers (LongROAD) project was a prospective cohort study of 2990 drivers aged 65-79 years at baseline and recruited between July 2015 and March 2017 from five sites (Ann Arbor, Baltimore, Cooperstown, Denver, and San Diego) in the contiguous United States. Study participants were assessed at baseline with the Patient-Reported Outcomes Measurement Information System (PROMIS). The objective of this study was to examine the prevalence and correlates of depression in older adults participating in the LongROAD project. Of the 2990 older adults studied, 186 (6.2%) participants had depression at the time of enrollment, with the lifetime prevalence of depression being 20.3%. Only 21.9% of the older adults with depression were taking antidepressants. A significantly higher prevalence of depression was found among those who were female (7.2%), not currently married (9.1%), with annual household income less than $20000 (11.9%), having diabetes (9.1%), having anxiety disorder (15.5%), with exercising routine (6.9%), and without doing volunteering work (7.9%). No significant difference was found between urban and rural residents, but urban residents had slightly higher depression prevalence (6.4%). About 1 in 16 older adults has depression. The majority (78%) of older adults with depression are not treated with antidepressants. Depression screening and treatment programs are needed for older adults, particularly women, those living alone, and those in poverty. Volunteering work could potentially decrease depression prevalence.

